# MiRNA-99a alleviates inflammation and oxidative stress in lipopolysaccharide-stimulated PC-12 cells and rats post spinal cord injury

**DOI:** 10.1080/21655979.2022.2031386

**Published:** 2022-02-08

**Authors:** Ruihong Wang, Yang Liu, Li Jing

**Affiliations:** a Department of Spine Surgery, Weifang People’s Hospital, Weifang, Shandong, China; b Department of Anesthesiology, Weifang People’s Hospital, Weifang, Shandong, China

**Keywords:** Spinal cord injury, miR-99a, NOX4, oxidative stress, cell apoptosis, inflammation, molecular mechanism

## Abstract

Spinal cord injury (SCI) is caused by spinal fracture after the displacement of the spine or broken bone fragments protruding into the spinal canal, resulting in different degrees of injury to the spinal cord or spinal nerves. Expression levels of miR-99a and nicotinamide adenine dinucleotide phosphate oxidase 4 (NOX4) in cerebrospinal fluid of SCI patients were analyzed. Rat adrenal gland pheochromocytoma cell line PC-12 were stimulated with lipopolysaccharide (LPS) to mimic the *in vitro* environment of SCI. A rat mode of SCI was established by laminectomy. Reactive oxygen species (ROS) levels were measured by 2’,7’-Dichlorodihydrofluorescein diacetate staining assay. Western blot was conducted to evaluate the expression levels of apoptotic indexes and proinflammatory cytokines. The interaction between miR-99a and NOX4 was verified by dual-luciferase reporter assay. The expression level of miR-99a was reduced while NOX4 expression was upregulated in cerebrospinal fluid of SCI patients and LPS-treated PC-12 cells. LPS impeded cell viability and promoted inflammation, apoptosis and ROS levels of PC-12 cells. Overexpression of miR-99a significantly promoted cell viability and reduced inflammation, apoptosis and oxidative stress in LPS-stimulated PC-12 cells. Dual-luciferase reporter assays verified that NOX4 was a target of miR-99a. Moreover, the expression of NOX4 was reduced in PC-12 cells after transfection with miR-99a mimic. Overexpression of NOX4 partly abolished the protective effect of miR-99a in LPS-treated PC-12 cells. To sum up, miR-99a suppresses NOX4 expression to relieve the LPS-induced inflammation, apoptosis and the progression of oxidative stress in SCI.

## Graphical Abstract

**Figure:**
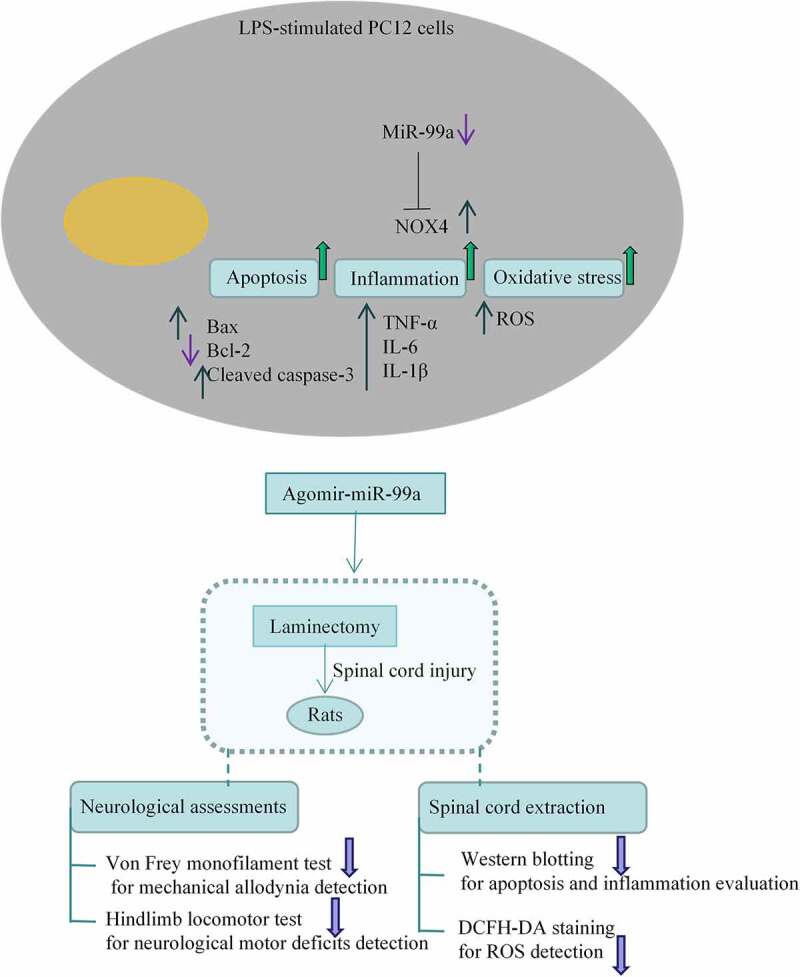

## Introduction

Spinal cord injury (SCI) is a trauma in the nervous system [1] including primary and secondary injuries. Primary injury is caused by mechanical compression. Secondary injury occurs after the primary injury, such as oxidative stress, post-traumatic inflammation and neuronal death [2]. Inflammation triggers the microglia activation, macrophage invasion, and nicotinamide adenine dinucleotide phosphate oxidase (NOX) production [3]. Inflammation is closely associated with oxidative stress [4]. In detail, oxidative stress triggers inflammation. Inflammation, in turn, enhances the production of reactive oxygen species (ROS). Oxidative stress exerts a crucial impact on secondary tissue damage and neuronal death in SCI. A recent study indicated that relieving oxidative stress can restrict spinal tissue injury in adult male mice post SCI [5].

NOX is a protease complex producing ROS. NOX4 belongs to the NOX family of oxidases, which is widely expressed and also exists in nerve cells [6,7]. NOX exhibits important effects in oxidative stress and inflammation in vasculature and central nervous system [8,9]. Oxidative stress leads to cell death and activates inflammation. Recent research has reported that inhibition of NOX4 suppresses NLR family pyrin domain containing 3 inflammasome activation in macrophages [7]. SCI can also induce the upregulation of NOX components [10].

MicroRNAs (miRNAs) are noncoding tiny RNAs that regulate physiological and pathological processes. They exert a key impact by post-transcriptional regulation [11]. Some of them are related to the inflammatory response and oxidative stress in SCI [12]. For example, miR-137 attenuates SCI by targeting neuronal differentiation 4 to reduce inflammation and oxidative stress [13]. MiR-146a relieves inflammation in the SCI model via blocking Toll-like receptor 4 signaling [14]. MiR-27b-3p can be used in gene therapy for treating SCI by regulation on microglia activation [15]. MiR-223 can alleviate the lipopolysaccharide (LPS)-induced PC-12 cell apoptosis and autophagy [16]. A previous study has indicated the reduced miR-99a level in rats post SCI [17]. Moreover, miR-99a can mitigate the LPS-induced inflammation of umbilical vein endothelial cells via inactivating the nuclear factor kappaB signaling [18]. It also mitigates oxidative injury in neuro-2a cells [19]. However, the mediatory role of miR-99a in SCI needs further study. We aimed to ascertain the role of miR-99a in LPS-treated PC-12 cells and in laminectomy-induced rat model of SCI by measuring apoptosis, inflammatiory response, and oxidative stress, thus providing insights into the potential targets for SCI treatment.

## Materials and methods

### Clinical samples

Sixty subjects in Weifang People’s Hospital were selected for investigation. Thirty patients with traumatic spinal fracture were defined as SCI group according to the American Spinal Injury Association (ASIA) criteria. Another 30 patients with limb fracture were enrolled as the control group. Their cerebrospinal fluids were collected. The written informed consent was signed by all subjects. All experimental procedures were approved by the Ethics Committee of Weifang People’s Hospital. This study is performed under full compliance with government policy and the Declaration of Helsinki.

### Cell culture and LPS treatment

The PC-12 cells were obtained from Procell (China). Cells (1 × 10^4^ cells/mL) were inoculated in Dulbecco’s modified Eagle medium with 10% fetal bovine serum (FBS; Procell, China), 100 U/mL penicillin, and 100 µg/mL streptomycin at 37°C under 5% CO_2_. The culture medium was changed three times a week. Cells were treated with LPS (0, 1, 5 and 10 μg/mL) for 12 h to induce the nerve injury model.

### Transfection

Lipofectamine 3000 (Thermo, USA) was used for transfection when the cells reached 80–90% confluence. MiR-99a mimic, miR-99a inhibitor and their negative controls (mimic control and inhibitor control) were ordered from GenePharma (China) and transfected into PC-12 cells. To determine the functions of NOX4, the coding regions of NOX4 were transfected into pEX-2 plasmids (GenePharma, China). All the trasnfections were performed at room tempetrature under a humidified asmotophere. Forty-eight h after transfection, the cells were harvested.

### Cell viability assay

Cell viability was detected using a Cell Counting Kit-8 (CCK-8; Solarbio, China). The cells were inoculated into 96-well plates with 5000 cells per well and left to attach overnight. After treatment with LPS, 20 μL of CCK-8 dissolved in serum-free medium was added into the plates for 2 h of culture at 37°C. The optical density were obtained by detecting absorbance at wavelength of 450 nm using a Microplate Reader (Reagen, China).

### Quantitative reverse transcription-polymerase chain reaction (qRT-PCR)

TRIzol (Invitrogen, USA) was applied to extract total RNA from 10^7^ cultured PC-12 cells or ~50 mg spinal cord tissues. The TaqMan MicroRNA Reverse Transcription Kit (AB, USA) was used for reverse transcription. SYBR Green Max Kit and ABI 7500 System were used for PCR amplification experiments. The thermal cycles are: 95°C for 5 min, 40 cycles of 95°C for 20s and 72°C for 10s. Glyceraldehyde-3-phosphate dehydrogenase (GAPDH) and U6 were used for normalization. The primer sequences were: miR-99a (F: 5’-AACCCGUAGAUCCGAUCUUGUG-3’, R: universal reverse primer); U6 (F: 5’-CAAGCAACGTTGTTTAA-3’, R: 5’-TAGTTAAACAACGTTGCTTC-3’); NOX4 (F: 5’-GGAGTTGACGTCGGAAT-3’, R: 5’-AGTAACTTCGACTTTAAGGT-3’); GAPDH (F: 5’-ACAACTTTGGTATCGTGGAAGG-3’, R: 5’-GCCATCACGCCACAGTTTC-3’). The relative expression levels were obtained via the 2^−ΔΔCT^ method [20].

### Western blot

Radio immunoprecipitation assay lysis buffer (Yeasen, China) was applied to extract proteins from PC-12 cells or spinal cord tissues. Protein concentration was determined with a bicinchoninic acid kit (Yeasen, China). Equal amounts of protein (30 μg) in all samples were separated using 12% sodium dodecyl sulfate polyacrylamide gel electrophoresis and transferred to polyvinylidene difluoride films (Yeasen, China). Nonspecific protein binding was blocked using 5% nonfat milk in 0.1% Tris Buffered Saline Tween for 2 h at room temperature. Next, the membranes were incubated with primary antibodies against interleukin (IL)-6 (1:1000, Abcam), IL-1β (1:1000, Abcam), tumor necrosis factor alpha (TNF-α; 1:1000, Abcam), B-cell lymphoma-2 (Bcl-2; 1:1000, CST), Bcl-2 associated X (Bax; 1:1000, CST), pro caspase-3 (1:1000, Abcam), cleaved caspase-3 (1:500, Abcam), NOX4 (1:2000, Abcam) and GAPDH (1:1000, CST) overnight at 4°C. Next, the horseradish peroxidase-marked secondary antibody was added and incubated for 1 h. The bands were visualized with the enhanced chemiluminescent kit (Yeasen, China). The expression levels of proteins were analyzed by the Image J software (version ImageJ 1.44P; National Institute of Health) to determine gray density.

### Terminal deoxynucleotidyl transferase-mediated dUTP nick end-labeling (TUNEL) assay

A TUNEL detection kit (Yeasen, China) was applied to determine cell apoptosis. Briefly, cells were fixed using 4% paraformaldehyde (Yeasen, China) and incubated with 0.3% Triton X-100 (Solarbio, China). Next, the TUNEL reaction mixture was applied to treat cells in the dark for 1 h, and apoptosis was detected by a fluorescence microscopy (Olympus, Tokyo, Japan). 4’,6-diamidino-2-phenylindole (DAPI; 1:30, Solarbio, China) was applied for nuclei staining for 3–5 min.

### Determination of ROS levels

The ROS level was evaluated using the 2’,7’-Dichlorodihydrofluorescein diacetate (DCFH-DA) staining with an ROS kit (Beyotime, China). The cells (1 × 10^5^/sample) were rinsed with PBS twice followed by centrifugation at 300 × g at 4°C for 5 min. Next, cells were incubated with 10 mM DCFH-DA at 37°C for 0.5 h. Next, the cells were observed under a fluorescent microscope (ex: 502 nm, em: 523 nm). Relative DCFH-DA fluorescence intensity was detected using a fluorometer.

### Dual-luciferase reporter assay

The targets of miRNA-99a were predicted using the TargetScan database (http://www.targetscan.org/vert_71/). Wild-type NOX4 3’-untranslated region (3’-UTR)(NOX4-WT) containing the predicted binding site of miR-99a was subcloned into the pmirGLO luciferase reporter vector (Progema). The mutant sequence was generated and named NOX4-MUT and was also inserted into the pmirGLO vector. Luciferase reporter vectors were cotransfected into PC-12 cells with miR-99a mimic or mimic control using Lipofectamine 3000. Forty-eight h later, the luciferase activity was examined using the dual luciferase reporter assay system(Progema). The firefly luciferase activity was normalized to renilla luciferase activity to eliminate the effects of inconsistent transfection effenciecy.

### Animals

Male Sprague-Dawley rats (Beijing Vital River Laboratory Animal Technology Co., Ltd., China) weighing 250–300 g at the age of 8–10 weeks were used in this study. Animals were housed in a controlled environment (25°C, 50% humidity, and 12-h light-dark cycle) and had free access to food and water. All experimental animals were randomly divided into the SCI-control, SCI-Agomir-NC, and SCI-Agomir-miR-99a groups with ten rats in each group. All experimental protocols were approved by the Animal Studies Ethical Committee at Weifang People’s Hospital.

### SCI model

Thoracic SCI was stimulated by laminectomy using a modified aneurysm clip (Mizuho, Tokyo, Japan) as previously reported [21,22]. The closing force was 30 g. After anesthetization using isoflurane, rats were placed in the prone position. Laminectomy was performed at the T6 and T7 vertebral segments using the T2 spinous process as a landmark. The aneurysm clip was applied extradurally to fully compress the spinal cord at the T6 level for 1 min. After surgery, rats were put on heated towels for recovery until they were fully awake. Bladders were manually pressed three times a day for urination until spontaneous reflexive bladder control was regained. On day 42, rats were euthanized and the spinal cord tissues were harvested.

### Injection of agomir-miR-99a

On day 1 post SCI, rats received injections of 0.5 nmol/μL agomir-miR-99a and agomir-NC (RiboBio) in the lesion epicenter using a microinjection pump and Neuros syringes at the rate of 0.2 μL/min. The needle was left in place for additional 5 min before being slow withdrawn.

### Neurological assessment

All neurological assessments were performed in a blinded way by two independent observers on days 1, 7, 14, 21, 28, 35, and 42 post SCI. Mechanical allodynia was evaluated using a Touch-Test 6-Piece Foot Sensory Evaluator Kit (Stoelting Co., Wood Dale, IL). Briefly, rats were placed on a rubber mat. The cutaneous pain threshold in response to graded mechanical pressure from 0.02 to 300 g on the dorsal surface of the trunk was tested using Von Frey hair. When rats showed consistent behavior of avoidance and vocalization over a relatively large skin area, the applied mechanical pressure were considered as the withdrawal thresholds. A single trial consisted of 12 applications of the von Frey filament at the interval of 2–3 seconds.

The Basso, Beattie, and Bresnahan (BBB) locomotor rating scale [23] was used to evaluate the neurological motor deficits, which has a range from zero to 21 points, judged by parameters such as tail balance, paw placement, and coordination of limb movement. Rats were observed for 5 min in the open field for evaluation of hindlimb locomotor function, and averaged values of individual scores were obtained.

### Statistical analysis

Experiments were repeated three times and the results were shown as mean ± standard deviation. Analyses were performed using the SPSS 19.0 software. Two-tailed, unpaired Student’s *t*-test or one-way analysis of variance were applied to compare the mean values between groups or among multiple groups, given that all data was normally distributed. *P* < 0.05 indicated a significant difference.

## Results

### The expression levels of miR-99a and NOX4 in SCI clinical samples

**Figure 1.**
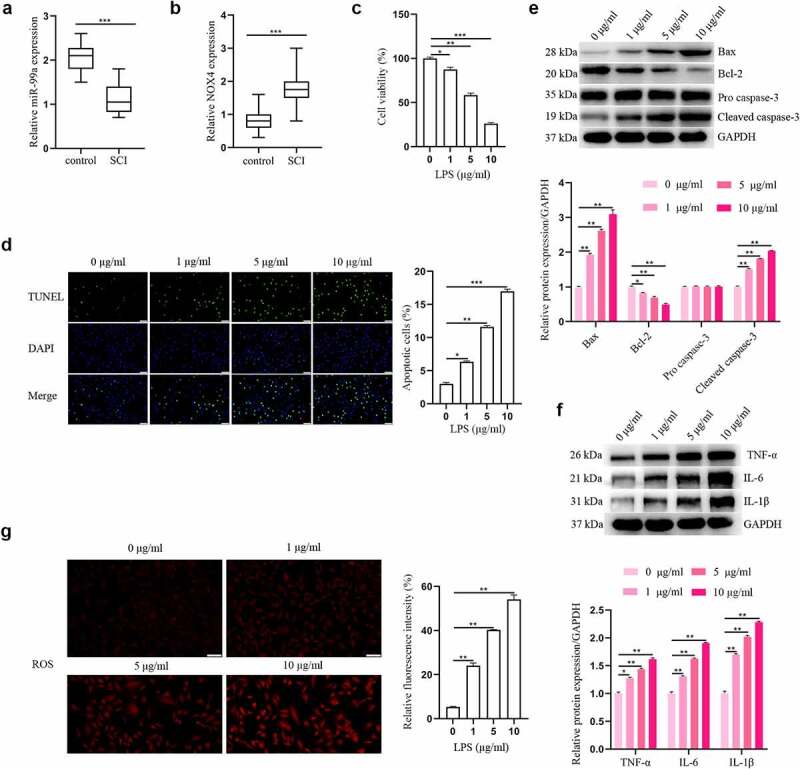
**The expressions of miR-99a and NOX4 in human cerebrospinal fluid samples and LPS-treated PC-12 cells**. The expressions of miR-99a (a) and NOX4 (b) in cerebrospinal fluid were tested using RT-qPCR analysis. (c) PC-12 cells were subjected to 0, 1, 5, 10 μg/ml LPS for 12 h, and cell viability was measured by CCK-8 assay. (d) Cell apoptosis was asessed using TUNEL assay. (e) Bax, Bcl-2, cleaved caspase-3 and pro caspase-3 protein expression levels were detected by Western blot. (f) The protein expression levels of TNF-α, IL-6 and IL-1β were estimated utilizing Western blot. (g) ROS was determined by DCFH-DA assay. Scale bars represent 50 μm. *p < 0.05, **p < 0.01, ***p < 0.001.

To reveal the clinical values of miR-99a and NOX4 in SCI, we first determined the levels of miR-99a and NOX4 in the cerebrospinal fluid of all patients with qRT-PCR. From the results, it was detected that the level of miR-99a in the SCI group was lower than the control group (t = 9.166, p < 0.001; Figure 1a). Furthermore, compared with the control group, the expression level of NOX4 was higher in the SCI group (t = 5.719, p < 0.001; Figure 1b).

### LPS triggered apoptosis, inflammation and oxidative stress in PC-12 cells

Effects of LPS on the apoptosis, inflammation, and oxidative stress in PC-12 cells were detected. After treating the PC-12 cells with different concentrations of LPS (1, 5 and 10 μg/mL), cell viability was detected. As shown in Figure 1c, LPS impeded cell viability in a dosage-dependent manner. Five μg/mL LPS was used for the subsequent assays. Figure 1d showed that LPS promoted PC-12 cell apoptosis in a dose-dependent manner. Meanwhile, the results of Western blot showed that LPS significantly enhanced Bax and cleaved caspase-3 expression levels, and reduced the level of Bcl-2 (Figure 1e). In addition, the effect of LPS on expression levels of inflammatory cytokines was explored. As depicted in figure 1f, the expression levels of IL-6, IL-1β, and TNF-α were all upregulated in PC-12 cells after LPS treatment in a dose-dependent manner. Moreover, LPS induced oxidative stress in PC-12 cells by increasing ROS secretion in a dose-dependent way (Figure 1g). Overall, these results indicated that LPS triggered apoptosis, inflammation and oxidative stress in PC-12 cells.

### MiR-99a protected PC-12 cells against LPS-induced injury by suppressing apoptosis, inflammation and oxidative stress

**Figure 2.**
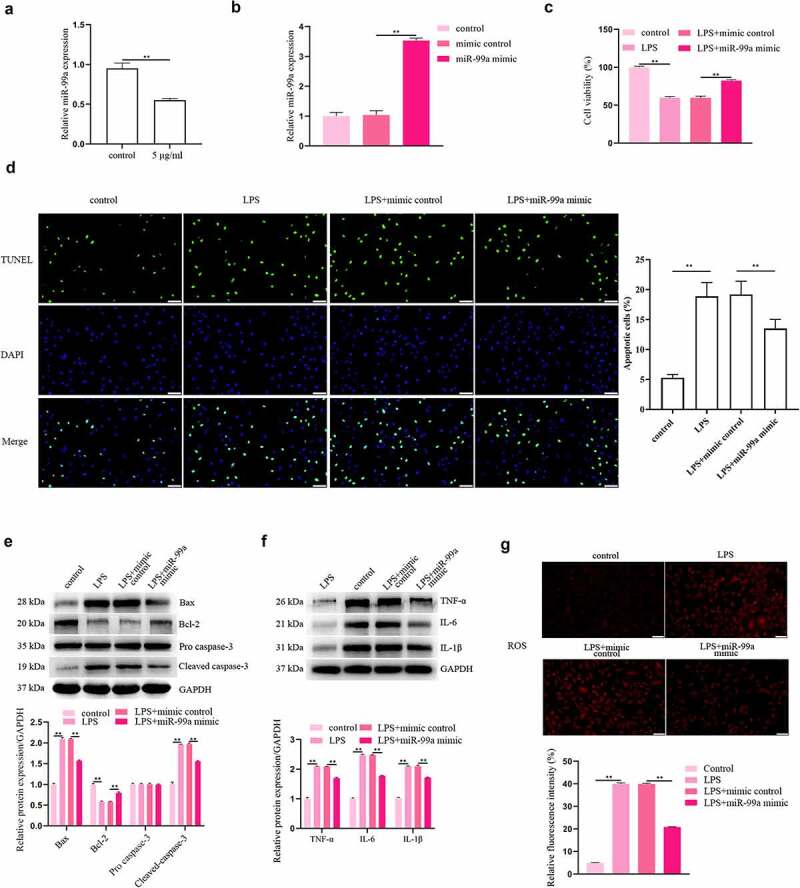
**MiR-99a played a protective role in LPS-treated PC-12 cells**. (a) MiR-99a level in LPS-stimulated PC-12 cells was tested using qRT-PCR analysis. (b) The level of miR-99a in PC-12 cells after transfection with miR-99a mimic or mimic control was measured by qRT-PCR. (c) After transfection with miR-99a mimic or mimic control, cell viability of LPS-treated PC-12 cells was measured by CCK-8 assay. (d) Cell apoptosis was measured by TUNEL assay. (e) Bax, Bcl-2, cleaved caspase-3 and pro caspase-3 protein levels were assessed by Western blot. (f) TNF-α, IL-6 and IL-1β protein levels were estimated by utilizing Western blot. (g) Effects of miR-99a mimic on ROS levels were determined by DCFH-DA staining assay. Scale bars represent 50 μm. **p < 0.01.

Whether miR-99a plays a role in the LPS-induced injury to PC-12 cells was explored. Based on the results of qRT-PCR, the level of miR-99a was markedly reduced in LPS-stimulated PC-12 cells (Figure 2a). Afterward, the level of miR-99a in PC-12 cells was significantly increased by transfection of miR-99a mimic, which indicated the successful transfection of miR-99a mimic (Figure 2b). The viability was detected with CCK-8 assay and the results showed that overexpression of miR-99a promoted the viability of LPS-treated PC-12 cells (Figure 2c). In addition, compared with the LPS + mimic control group, transfection with miR-99a mimic decreased cell apoptosis rate (*p* < 0.05, Figure 2d). Protein expression levels of Bax and cleaved caspase-3 were decreased while Bcl-2 expression was increased by miR-99a mimic in LPS-stimulated PC-12 cells (Figure 2e). Furthemore, transfection of miR-99a mimic also significantly decreased the expression levels of IL-6, IL-1β, and TNF-α in LPS-treated PC-12 cells (figure 2f). Intracellular ROS levels were markedly reduced in LPS + miR-99a mimic group compared with the LPS + mimic control group (Figure 2g).

### MiR-99a improves allodynia and functional motor recovery and suppresses apoptosis, inflammation, and oxidative stress in rats post SCI

**Figure 3.**
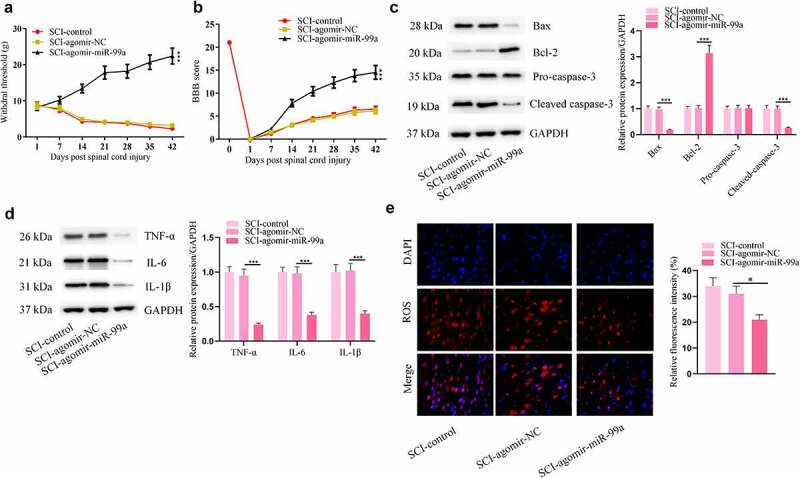
**MiR-99a improved allodynia and functional motor recovery and suppressesd apoptosis, inflammation, and oxidative stress in rats post SCI**. (a) Cutaneous pain threshold of rats in the SCI-control, SCI-Agomir-NC, and SCI-Agomir-miR-99a groups was evaluated by von Frey filament test. (b) BBB score of the rats. (c) Bax, Bcl-2, cleaved caspase-3 and pro caspase-3 protein levels in the spinal cord tissues were assessed by Western blot. (d) TNF-α, IL-6 and IL-1β protein levels in the spinal cord tissues were estimated by Western blot. (e) ROS levels in the spinal cord tissues were determined by DCFH-DA staining assay. *p < 0.05, **p < 0.01, ***p < 0.001.

Impacts of miR-99a in rats post SCI were further investigated. Agomir-miR-99a-treated rats showed significant improvement in neuropathic pain threshold, as detected by the von Frey monofilament test (Figure 3a). These animals showed better functional motor recovery than control animals (Figure 3b). After injection with agomir-miR-99a, apoptosis and inflammation in the spinal cord were suppressed, as indicated by the decrease of BAX, cleaved caspase-3, TNF-α, IL-6, IL-1β levels and the increase of Bcl-2 levels (Figure 3c-D). Moreover, miR-99a resuced ROS levels in the spinal cord of rats after SCI (Figure 3e).

### MiR-99a can sponge NOX4

**Figure 4.**
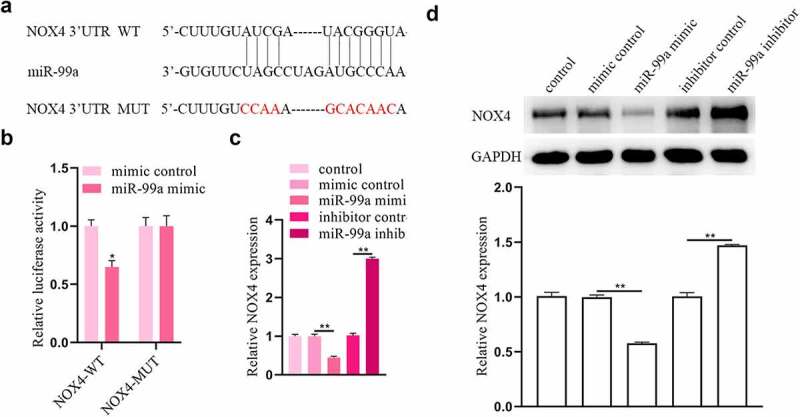
**NOX4 was a target gene of miR-99a**. (a) The binding site of miR-99a on the 3’-UTR of NOX4 was predicted from Targetscan. (b) Luciferase reporter assay revealed the binding relationship between miR-99a and NOX4 3’-UTR. (c) The expression level of NOX4 in LPS-treated PC-12 cells after transfection with miR-99a mimic, mimic control, miR-99a inhibitor and inhibitor control was measured by qRT-PCR. (d) The protein expression level of NOX4 was estimated by Western blot. *p < 0.05, **p < 0.01.

Furthermore, the targets of miR-99a were explored. Using TargetScan, we discovered that miR-99a can interact with the 3ʹUTR of NOX4 (Figure 4a). The luciferase reporter assay was conducted to confirm their binding relationship. It was revealed that the luciferase activity of NOX4-WT in PC-12 cells was suppressed by cotransfection with miR-99a mimic, while that of NOX4-MUT was not significantly affected by miR-99a mimic (Figure 4b). In addition, transfection of miR-99a mimic decreased the mRNA and protein expression levels of NOX4 in PC-12 cells (Figure 4c and 4D). On the contrary, transfection with miR-99a inhibitor had opposite effects on NOX4 expression levels.

### NOX4 participated in the process of miR-99a-mediated protection in LPS-treated PC-12 cells

**Figure 5.**
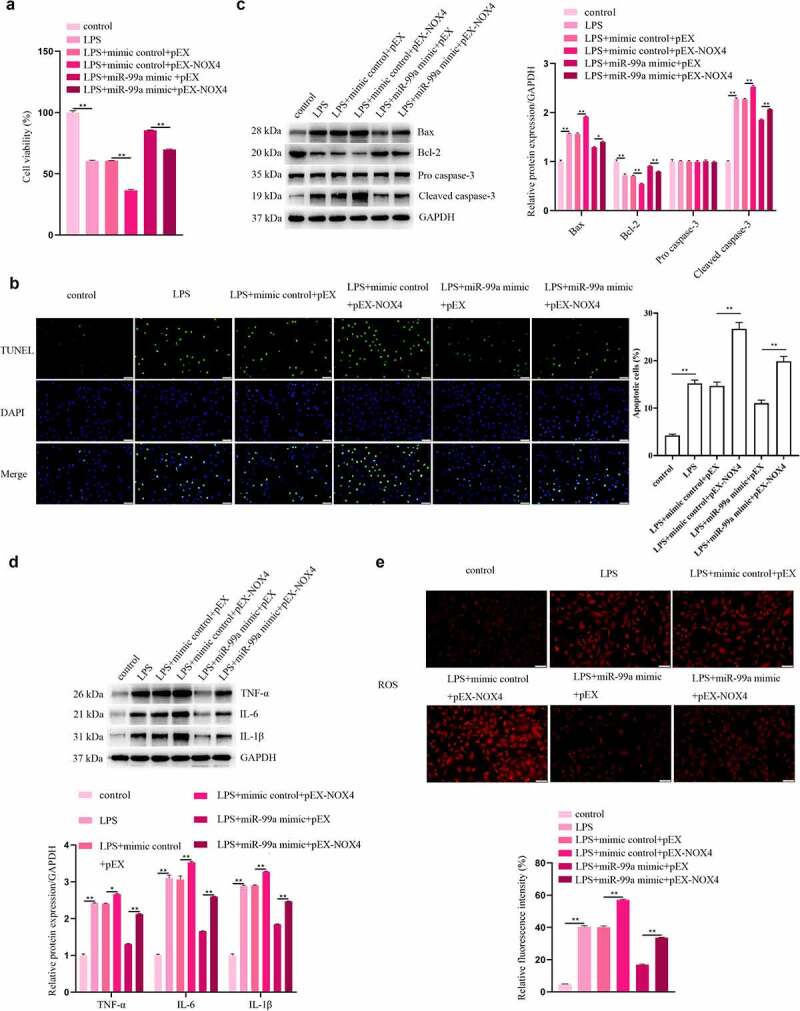
**NOX4 participated in the regulatory effect of miR-99a on LPS-treated PC-12 cells**. (a) Viability of LPS-treated PC-12 cells after transfection with miR-99a mimic, mimic control, pEX-NOX4 and control empty pEX vector was measured by CCK-8 assay. (b) Cell apoptosis was assessed by TUNEL assay. (c) Bax, Bcl-2, cleaved caspase-3, and pro caspase-3 protein expression levels were estimated by Western blot. (d) TNF-α, IL-6 and IL-1β protein expression levels were estimated by Western blot. (e) ROS level was determined by DCFH-DA staining assay. Scale bars represent 50 μm. *p < 0.05, **p < 0.01.

To investigate the roles of NOX4 in LPS-stimulated PC-12 cells, the cells were cotransfected with miR-99a mimic and pEX-NOX4, and then treated with LPS. As shown in Figure 5a-E, NOX4 overexpression decreased viability and aggravated apoptosis, inflammation and oxidative stress in LPS-treated PC-12 cells. Moreover, NOX4 rescued the miR-99a-mediated increase of viability and decrease of apoptosis, inflammatory response, and oxidative stress in LPS-stimulated PC12 cells. The findings indicated that miR-99a alleviated the LPS-induced injury in PC-12 cells by targeting NOX4.

## Discussion

SCI is a traumatic neuronal injury that threatens human health. It has been discovered that TNF-α and IL-6 levels were raised after spinal cord contusion in rats. The inflammatory reactions under abnormal conditions trigger cell apoptosis [24]. LPS has been widely used to induce cell injury [25]. In the present research, our results presented that LPS impaired PC-12 cell viability, increased apoptosis, accumulated intracellular ROS, and enhanced the productions of inflammatory cytokines.

MiRNAs mediate the development and function of neurons [26]. They have shown mediatory influences in spinal cord diseases. It has been manifested that the IL-7-induced inflammation can be reduced by downregulating miR-136-5p expression through the nuclear factor kappaB signaling [27]. MiR-214-3p and miR-211 repair spinal cord injury [28,29]. According to the research, we find that in SCI, the level of miR-99a is decreased [30]. MiR-99a targets TNF-α to suppress M1 macrophage phenotype and inflammation [31] and ameliorates the LPS-induced injury by activating the Notch pathway in H9c2 cells [32]. Furthermore, miR-99a suppresses the apoptosis of cardiomyocytes in ischemia via reducing the expression of cleaved caspase-3 [33]. Here, we reported that miR-99a increased neuronal viability, decreased apoptosis, suppressed intracellular ROS levels, and inhibited secretion of proinflammatory cytokines in the LPS-treated PC12 cells. Moreover, miR-99a improved mechanical allodynia and functional motor recovery and suppressesd apoptosis, inflammation, and oxidative stress in rats post SCI.

The NOX family is a primary source of ROS and oxidative stress [34]. The activated NOX4 utilizes oxygen as a substrate to promote ROS production. It contributes to OGD/R-induced neuronal apoptosis [35]. Suppression of NOX4/ROS alleviates neuronal and blood-brain barrier injury via reduction of oxidative stress after intracerebral hemorrhage [36]. In addition, the use of NOX inhibitor apocynin presents the anti-inflammatory function against stroke [37]. In SCI, reduction of NOX2 can inhibit inflammation and oxidative stress [5]. Herein, we found that the expression of NOX4 was upregulated in cerebrospinal fluid samples of SCI patients. We predicted the target genes of miR-99a through the bioinformatics and found that NOX4 was a potential target. Moreover, the expression of NOX4 was reduced by miR-99a mimic and upregulated by miR-99a inhibitor in LPS-treated PC-12 cells. It was also found that overexpression of NOX4 restricted the protective effects of miR-99a on LPS-induced PC-12 cell injury. All the results suggested that miR-99a plays a protective role in the recovery of SCI and alleviates nerve injury by targeting NOX4 to relieve apoptosis, inflammation and oxidative stress.

## Conclusion

MiR-99a ameliorates the LPS-induced cell injury through targeting NOX4. MiR-99a improves allodynia and functional motor recovery and suppresses apoptosis, inflammation, and oxidative stress in rats post SCI. Our results provides a reliable basis for exploring the role of miR-99a in SCI and indicates miR-99a as a new target for the clinical treatment of SCI.

## Data Availability

The analyzed data sets generated during the present study are available from the corresponding author on reasonable request.

## Abbreviation

ROS, reactive oxygen species; SCI, spinal cord injury; CNS, central nervous system; PVDF, Polyvinylidene difluoride; OD, Optical density; SD, Standard deviation

## Highlights

1. MiR-99a reduces apoptosis, inflammation, oxidative stress in LPS-treated PC-12 cells.

2. NOX4 is a target of miR-99a.

3. MiR-99a improves mechanical allodynia and functional motor recovery in SCI rats.

4. MiR-99a suppresses apoptosis, inflammation, and oxidative stress in rats post SCI.
